# Single-Port Trans-Perineal Approach to Cystoprostatectomy with Intracorporeal Ileal Conduit Urinary Diversion and Lymph-Nodes Dissection using a Purpose-Built Robotic System: Surgical Steps in a Preclinical Model

**DOI:** 10.1590/S1677-5538.IBJU.2018.0524

**Published:** 2019-09-02

**Authors:** Juan Garisto, Riccardo Bertolo, Eddie Chan, Jihad Kaouk

**Affiliations:** 1 Glickman Urological & Kidney Institute, Cleveland Clinic, Cleveland OH, USA;>; 2 Chinese University of Hong Kong and Division of Urology at Prince of Wales Hospital, Shatin, Hong Kong

## Abstract

**Aim:**

To report the technique for single-port trans-perineal cystoprostatectomy with intracorporeal ileal conduit urinary diversion and lymph nodes dissection using a purpose-built robotic platform (da Vinci SP1098, Intuitive Surgical, Sunnyvale, CA, USA).

**Materials and Methods:**

In a male cadaver the SP1098 robotic system was used to perform cystoprostatectomy with intracorporeal ileal conduit urinary diversion and lymph nodes dissection by single-port trans-perineal approach. The surgery was completed through a 2.5-cm perineal incision through which a GelPOINT Mini advanced access platform (Applied Medical, Rancho Santa Margarita, CA, USA) and a dedicated 25-mm multichannel port accommodating a 12 x 10-mm oval articulating robotic camera, three 6-mm double-jointed articulating robotic instruments and a 6-mm accessory laparoscopic instrument were positioned. At the planned level of the stoma for the ileal conduit, a 12-mm port was placed through which the EndoGIA® stapler was used to mature the urinary diversion

**Results:**

The total operative time was 185 min. The procedure was successfully completed without the need for additional ports placement. The benefits of the trans-perineal approach, particularly in longer procedures as radical cystectomy with intracorporeal urinary diversion, might include the avoided need of Trendelenburg position, with undoubtful advantages for the patient and the anesthesiologist in terms of respiratory mechanics and hemodynamics.

**Conclusions:**

The feasibility of single-port trans-perineal cystoprostatectomy with intracorporeal ileal conduit urinary diversion and lymph nodes dissection using the SP1098 purpose-built robotic platform is demonstrated. The duplication of the described surgical steps in the clinical model is awaited when the platform will be available on the market.

**ARTICLE INFO**

*Riccardo Bertolo*

http://orcid.org/0000-0003-0260-4601

Available at: http://www.intbrazjurol.com.br/video-section/20180524_Garisto_et_al

Int Braz J Urol. 2019; 45 (Video #15): 854-5

